# New Insights into the Implication of Mitochondrial Dysfunction in Tissue, Peripheral Blood Mononuclear Cells, and Platelets during Lung Diseases

**DOI:** 10.3390/jcm9051253

**Published:** 2020-04-26

**Authors:** Marianne Riou, Abrar Alfatni, Anne-Laure Charles, Emmanuel Andrès, Cristina Pistea, Anne Charloux, Bernard Geny

**Affiliations:** 1 University of Strasbourg, Translational Medicine Federation of Strasbourg (FMTS), Faculty of Medicine, Team 3072 “Mitochondria, Oxidative Stress and Muscle Protection”, 11 rue Humann, 67000 Strasbourg, France; marianne.riou@chru-strasbourg.fr (M.R.); aaalfatni@hotmail.com (A.A.); anne.laure.charles@unistra.fr (A.-L.C.); cristina.pistea@chru-strasbourg.fr (C.P.); anne.charloux@chru-strasbourg.fr (A.C.); 2 University Hospital of Strasbourg, Physiology and Functional Exploration Service, 1 Place de l’Hôpital, CEDEX, 67091 Strasbourg, France; 3 Internal Medicine, Diabete and Metabolic Diseases Service, University Hospital of Strasbourg, 1 Place de l’Hôpital, CEDEX, 67091 Strasbourg, France; emmanuel.andres@chru-strasbourg.fr

**Keywords:** lung diseases, mitochondria, blood, PBMCs, platelets, oxidative stress, COPD, asthma, pulmonary arterial hypertension, pulmonary fibrosis

## Abstract

Lung diseases such as chronic obstructive pulmonary disease, asthma, pulmonary arterial hypertension, or idiopathic pulmonary fibrosis are major causes of morbidity and mortality. Complex, their physiopathology is multifactorial and includes lung mitochondrial dysfunction and enhanced reactive oxygen species (ROS) release, which deserves increased attention. Further, and importantly, circulating blood cells (peripheral blood mononuclear cells-(PBMCs) and platelets) likely participate in these systemic diseases. This review presents the data published so far and shows that circulating blood cells mitochondrial oxidative capacity are likely to be reduced in chronic obstructive pulmonary disease (COPD), but enhanced in asthma and pulmonary arterial hypertension in a context of increased oxidative stress. Besides such PBMCs or platelets bioenergetics modifications, mitochondrial DNA (mtDNA) changes have also been observed in patients. These new insights open exciting challenges to determine their role as biomarkers or potential guide to a new therapeutic approach in lung diseases.

## 1. Introduction

Lung diseases, especially chronic obstructive pulmonary disease (COPD), asthma, pulmonary arterial hypertension (PAH), and/or idiopathic pulmonary fibrosis (IPF) are main causes of mortality, resulting in significant health and economic burdens worldwide. Indeed, the prevalence of COPD is increasing with 64 million persons concerned in the world, and according to the latest WHO estimates, it will rise as the third leading cause of death by 2030 [1]. Asthma is the most common chronic disease among children, affecting around 235 million persons [2]. PAH or IPF are less common but are characterized by poor prognosis [3,4]. Based on this evidence, it appears important to gain new insight into lung diseases pathophysiology and to analyze potential biomarkers to better reveal lung function, diagnose, and predict the prognosis of these diseases. As observed during cardiovascular diseases, mitochondrial dysfunctions deserve to be further studied, both at the local and at the circulating levels [5]. Indeed, mitochondrial abnormalities are involved in lung diseases but direct evidence of mitochondrial dysfunction in human studies is limited, because of the difficulty to biopsy tissues in these frail patients.

Peripheral blood mononuclear cells (PBMCs) or platelets are accessible through a small amount of blood withdrawal and allow mitochondrial function analysis. PBMCs are composed by lymphocytes, monocytes, and dendritic cells, which mainly participate in immunity and inflammation. Platelets are known to modulate hemostasis and are also involved in pulmonary alterations. Isolation of circulating leukocytes is an easy way to represent cardiovascular stress [6,7,8]. Recent studies in sepsis have suggested that bioenergetics profiling of circulating PBMCs might reflect mitochondrial function in other tissues and are linked with disease grade, immune alterations, and prognosis [9,10,11,12]. At rest, PBMCs rely mainly on mitochondrial respiration to match the metabolic demand and show a significant spare respiratory capacity [13]. Compared to biopsy, blood withdrawal is easy, but isolating purified platelets is a time-consuming task and analyzing mitochondria from these platelets needs to be done in a timely and skillful manner [14]. Circulating platelets are rich in mitochondria and could be used to assess bioenergetics and systemic metabolism in pathologies such as diabetes, sepsis, and cardiovascular or sickle cell diseases [15,16,17]. Reactive oxygen species (ROS) is an important factor in platelet functioning [18], as observed in COPD and adult respiratory distress syndrome [19,20].

The mean bioenergetics profiles in human circulating platelets, monocytes, lymphocytes, and neutrophils were reported in real-time measurements of the mitochondrial oxygen consumption rate (OCR) using the extracellular flux analyzer (Seahorse Bioscience) [21]. In a healthy subject, monocytes are one of the most energetic cell types with high levels of both glycolysis and oxidative phosphorylation. Lymphocytes and platelets are more oxidative and less glycolytic at the basal state; neutrophils show little mitochondrial oxidative capacities [21]. The reserve capacity, difference between basal and maximal mitochondrial respiration, is potentially used by cells in the setting situations needing higher mitochondrial involvement and is about 20% in platelets, whereas it is highest in monocytes or lymphocytes. 

**Figure 1:**
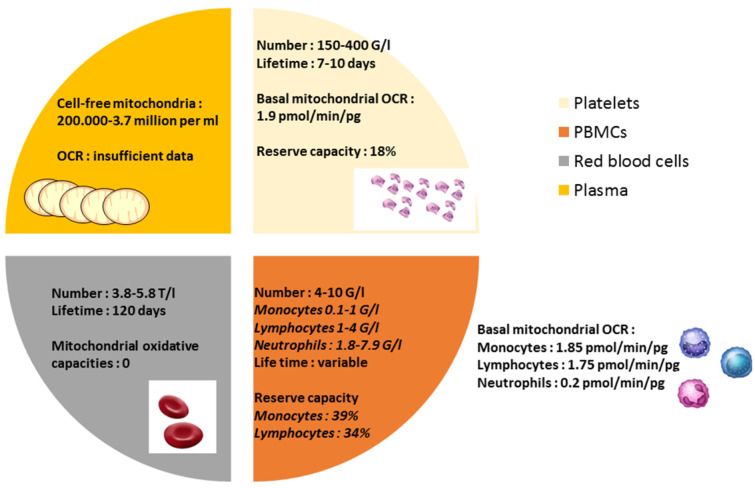
Characteristics of circulating blood cells and plasma: number, lifetime, and mitochondrial oxidative capacity. OCR: mitochondrial oxygen consumption rate.

Interestingly, despite red blood cells being present with no mitochondria in human, a recent report showed a presence of structurally cell-free competent mitochondria in blood circulation [22]. Circulating peripheral blood cells and plasma characteristics and bioenergetics are presented in Figure 1.

The study of mitochondrial function in PBMCs and circulating platelets seems to be useful and complementary [13,21]. At present, the link between PBMCs or platelets mitochondrial function and lung diseases is poorly known and the objective of this review is to present the data published so far and to discuss the potential interest of studying circulating blood cells mitochondrial function and ROS production during COPD, asthma, PAH, and IPF.

## 2. Physiological Mitochondrial Function

Derived from ancient aerobic bacteria, mitochondria are double membrane structures with its own maternally DNA and transcription machinery. Mitochondria are involved in the heme biosynthesis, intracellular calcium regulation, ATP-production, and fatty acid synthesis. Mitochondria are the main source of energy in the cells and oxygen consumption by the mitochondrial respiratory chain (electron transport system—ETS) drives adenosine triphosphate (ATP) synthesis. Defects of mitochondria result in an energy deficiency that can impair cells, and lately the entire organism function. The mitochondrial respiratory system is located in the inner mitochondrial membrane and is formed by five complexes (I-IV and complex V–ATP synthase). Each complex can be analyzed with a spectrophotometer and, besides oxygen consumption, ATP synthesis and mitochondrial membrane potential can be determined [23,24]. Importantly, mitochondrial also generate ROS (i.e., anion superoxide arising from complexes I and III and dismutated to hydrogen peroxide by superoxide dismutase in hydrogen peroxide, which is able to leave the mitochondria), considered as signaling molecules at normal levels but able to generate proteins, lipids, and nucleic acids damages at higher levels [25]. 

Mitochondria contain DNA (mtDNA) and their own transcriptional mechanisms. MtDNA is very sensitive to oxidative stress and genotoxic agents because of its proximity to the mitochondrial respiratory chain, the major site of ROS production. If mtDNA is damaged, an increasing number of mtDNA copies appears as compensation and is believed as an indicator of mitochondrial function and oxidative stress [26]. After injury, mtDNA fragments accumulate in autolysosomes and are partially degraded by DNAse II.

## 3. Mitochondrial Dysfunction in Lung Diseases

Many of the lung diseases are thought to be related to aging, and accumulation of dysfunctional mitochondria is considered a marker for the pathological conditions but is also the key factor that drives disease progression. Thus, mitochondrial dysfunction may contribute to the pathogenesis of many human diseases including lung diseases such as COPD, asthma, PAH, and IPF [27,28], often associated with increased ROS and impaired bioenergetics and/or mitochondrial biogenesis, mitophagy, and dynamic, which are critical to maintain cell homeostasis and may result in cellular apoptosis and senescence [29]. Lungs are exposed to the high-oxygen environment and present huge contact areas with the blood to allow hematosis, making them sensitive to oxidative stress and damages. For example, tobacco smoking, a major risk factor for lung diseases, is linked to oxidative stress and induces mitochondrial dysfunction [30]. Mitochondrial ROS can favor pro-inflammatory cytokines secretion and modulate calcium regulation of epithelium and airway muscle cells or extracellular matrix production [31]. Lung parenchymal and immune cells communicate in response to infections, cigarette smoke, and air pollution etc., in order to repair tissues or defend against pathogen, but data on circulating blood cells are sparse. 

In addition, some studies have suggested that mtDNA fragments could be implicated in lung diseases pathophysiology, acting as a damage-associated molecular pattern (DAMP) to initiate immunological response [32].

### 3.1. Chronic Obstructive Pulmonary Disease (COPD)

#### 3.1.1. Mitochondrial Dysfunction and Oxidative Stress in COPD

COPD is a major cause of respiratory failure with a prevalence of 10% in adults over 40 years [1]. The estimates show that it will be the third cause of mortality in 2030. The pathogenesis of COPD is not completely understood. Chronic inflammation is a central feature, leading to airway remodeling, irreversible bronchial obstruction, and destruction of lung parenchyma (emphysema). Acute exacerbations, whatever their origin, increase oxidative stress and induce systemic inflammation both in lung resident and circulating cells of individual patients [33]. Oxidant–antioxidant imbalance is one of the involved mechanisms, but how mitochondrial mediated-inflammation contributes to the disease progression remains to be determined. Airway epithelium is very sensitive to oxidative stress because of a low expression of anti-oxidative enzymes which are sparsely inducible, mainly after tobacco exposure [34]. In COPD, oxidative stress is enhanced particularly after exacerbation. At the systemic level, plasmatic ROS are enhanced in smokers, whether or not with COPD, and oxidative capacity is lower in plasma of smokers. Wiegman et al. showed an association between mitochondrial dysfunction and excessive mtROS levels in airway smooth muscle cells from COPD patients, which contributes to enhanced inflammation and cell proliferation [31].

In COPD, mtROS are released from activated inflammatory cells or structural cells such as epithelial, endothelial, or smooth muscle cells [35]. In this way, oxidative stress is an adaptive response. It is a mechanism leading to initiate immune response to neutralize infectious agents and to maintain the cellular homeostasis [36]. In excess, ROS damages DNA, lipids, and proteins, resulting in lung cellular death, activation of metalloproteases, inactivation of antiproteases, and degradation of extracellular matrix which result in loss of alveolar units [37]. ROS participate also in cell proliferation and collagen synthesis in smooth muscle.

Moreover, ROS activate redox sensitive transcription factors such as nuclear factor-kappa B (NFκB), resulting in release of pro-inflammatory mediators such as IL 1-like cytokines [36,38]. Levels of IL-1β are increased in lungs of patients with COPD after smoking, suggesting the involvement of inflammasome [38,39,40].

#### 3.1.2. Mitochondrial Function, Oxidative Stress, and mtDNA in PBMCs or Platelets in COPD

PBMCs have been shown to release more ROS, which may contribute to COPD patient’s prognosis [41]. A recent study observed a high level of mitochondrial dysfunction and derived ROS in PBMCs obtained from unstable COPD patients after combustion-generated ultrafine particles exposure [42]. Indeed, exposure to nano-organic carbon particles and soot ultrafine particles (found in environmental pollution) induced the release of pro-inflammatory IL-18 and IL-33 from exacerbated COPD-derived PBMCs, with oxidative stress. Further, a recent work observed that PBMCs in COPD have reduced ability to use glucose, pyruvate, or fatty acids at baseline, which is not observed in PBMCs from healthy smokers which have only impaired glycolysis [43]. Similar results have been observed in sepsis [44].

Because COPD is frequently associated with cardiovascular diseases, activation of blood platelets through inflammation seems to have an important role in the pathophysiology of COPD [19]. After respiratory exacerbation, thrombocytosis is associated with significantly short and long term mortality, supporting an important role of platelets [45]. Antiplatelet therapy may have a protective role in patients after exacerbation of COPD. A study has shown that, during COPD, hypercoagulation (measured through platelet distribution width) is associated with reduced survival [46]. In a guinea pig model, Bialas et al. showed increased proton and electron leaks and decreased mitochondrial respiratory chain capacity in platelets from chronic smoke-exposed animals [47]. Proton and electron leaks in platelets appeared related to ROS production and interactions between oxidative stress and platelets could represent potential therapeutic targets. Indeed, administration of N-acetylcysteine improved the quality of life of stable COPD patients [48].

MtDNA levels can be modulated by oxidative stress in COPD, as observed in the exhaled breath or urine [49,50]. In leukocytes, mtDNA easily undergo mutations, insertions, or deletions in response to oxidative stress during COPD. Liu et al. showed a decreased peripheral leukocyte mtDNA copy number in COPD patients, suggesting a less mtDNA protection or biosynthesis in these patients [51]. Mitochondrial dysfunction could result in abnormal function of leukocytes in COPD. In this study, the mtDNA copy number was similar in healthy smokers and non-smokers subjects. On the contrary, a study showed an increase of mtDNA/nuclear DNA ratio in the blood from patients with ACOS (asthma-COPD overlap syndrome). Interestingly, there was a correlation between mtDNA and the number of smoked cigarettes [52]. The increase of mtDNA content could compensate the mitochondrial respiratory function decline due to oxidative damage or mutation in this pathology.

In addition, Kim et al. demonstrated that peripheral leukocyte mtDNA copy numbers positively correlated with leukocytes telomeres length in elderly women, suggesting that telomere may relate to mitochondrial function [53]. Indeed, COPD patients have short leukocyte telomeres, associated with an increased risk of total and cancer mortality [54,55].

**Figure 2:**
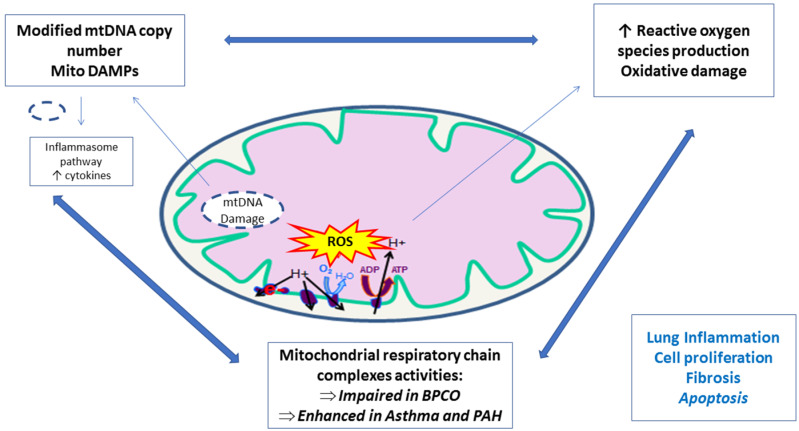
Peripheral blood mononuclear cells and platelets mitochondrial implication in lung diseases.

In summary, mitochondrial dysfunctions in PBMCs of COPD patients result likely in abnormal functionality, and mitochondrial pathophysiology represents an emerging research with potential promising therapeutic avenues (Table 1, Figure 2, modified from [56]).

### 3.2. Asthma

#### 3.2.1. Mitochondrial Function and Oxidative Stress in Asthma

Asthma is a frequent disease that affects around 235 million persons [2], showing airflow obstruction, bronchial hyper-reactivity, and inflammation [57]. It is multifactorial, favored by individual/genetic and general/environmental parameters. The progression of bronchial epithelium damage is related to increased inflammation, favored by cytokines release. Mitochondrial dysfunction and enhanced oxidative stress participate in the pathophysiology of asthma through increased mucus secretion and impaired bronchial smooth muscles, as observed both in experimental and clinical studies [58,59,60,61,62,63,64]. Accordingly, a decreased antioxidative capacity inferred from reduced superoxide dismutase, gluthation peroxidase, or catalase activity was related to the gravity of asthma [34,61,62,63]. Interestingly, the role of ROS is not unequivocal depending on the cells involved. Thus, if it is generally accepted that ROS arising from epithelial and smooth muscle cells participate in lung injury and aggravate inflammation [64], ROS might be protective when present in the blood. 

#### 3.2.2. Enhanced Mitochondrial Function and ROS Production in PBMCs or Platelets in Asthma

Currently, during asthma, there are some data on the mitochondrial implication of peripheral blood circulating cells. In school-aged children with atopic asthma, antigen-specific IgE receptor expression was revealed on PBMCs [65]. In addition, future exacerbations were associated with the number of basophils during childhood acute wheeze/asthma [66]. Recently, Ederle et al. showed, in severe exacerbated asthmatic patients, an enhanced PBMCs mitochondrial respiration and increased ROS production compared to healthy volunteers [67]. Interestingly, the plasma of asthmatic patients stimulated similarly the PBMC’s of control subjects, suggesting a mechanism of protection as proposed during septic shock [68]. On the contrary, PBMCs impaired mitochondrial function was observed early (6 hours) in patients with local allergic rhinitis after an acute nasal allergen challenge [69]. Likewise, PBMCs oxidative capacity was often reduced in cardiovascular diseases [7]. In the study of Ederle et al., obesity might have played a role. Indeed, PBMCs mitochondrial respiration tend to be enhanced in asthmatic patients presenting with a BMI ≥30 kg/m^2^ [67] and differential bioenergetics in airway epithelial cells and platelets between lean and obese asthmatics was observed [70,71]. Of note, steroids mediate eosinophils apoptosis via a mitochondrial pathway, and such a link needs to be further studied since systemic steroid treatment longer than 24 hours did not influence the ROS production [72]. 

Increasing evidence suggests an important participation of platelets and their secretions (thromboxane, serotonin…) in the pathophysiology of allergic diseases and a potential role as key modulators of immunity. In patients with asthma, alterations in platelets secretion, expression of surface molecules such as IgE receptors, aggregation, and adhesion were observed [73,74]. Platelets activation in bronchoalveolar lavage was associated with airway hyperreactivity [75,76,77]. Platelets contribute also to the secretion of cytokines and can activate eosinophils [78,79]. Xu et al. showed increased Krebs cycle enzyme activity and less dependence on glycolysis in platelets of asthmatic patients [80]. Taken together, these data suggest a capacity for greater oxygen consumption and more efficient energy production in platelets of asthmatic patients (Table 2).

### 3.3. Pulmonary Hypertension

#### 3.3.1. Mitochondrial Dysfunction, ROS, and mtDNA in Pulmonary Hypertension

Pulmonary hypertension (PH) is classified in five groups, depending on hemodynamic characteristics [83,84]. Post-capillary PH (group 2) is secondary to left heart diseases [83]. In left heart diseases, PBMCs undergo changes similar to failing cardiomyocytes and the degree of PBMCs mitochondrial dysfunction and increased ROS can be related to the disease severity [6,7,8,85,86,87]. Classically, group 1 of PH, which includes idiopathic pulmonary arterial hypertension (PAH), is characterized by pulmonary artery remodeling with intimal fibrosis, medial smooth cells hypertrophy, and in situ thrombosis with plexiform lesions. This arterial remodeling is associated with elevated pulmonary vascular resistance, potentially leading to right ventricular failure and death [88] and we can expect a potential implication of mitochondrial impairment and ROS in PAH.

Particularly, they favor vessels constriction progression through wall thickening via growth factor stimulation and endothelin-1-related smooth muscle proliferation [89,90,91,92,93]. Hypoxic response, inflammation, apoptosis, and vasoconstriction in PAH might also use endothelial mitochondria pathways. In animals and humans, endothelial and pulmonary arterial smooth muscle cells exhibit metabolic switch from mitochondrial oxidative phosphorylation toward cytoplasmic glycolysis even in the presence of oxygen, conferring apoptosis resistance and cellular hyper proliferation [94,95]. This is accompanied by altered mitochondrial ETC activity [94]. These abnormalities concern also cardiac tissue and cells as well as skeletal muscle of PH patients [96,97,98,99,100,101]. Further, altered *BMPR2* expression, gene implicated in PAH predisposition, was linked to pulmonary arterial endothelium mitochondrial dysfunction [102] and ROS upregulate hypoxia-inducible transcription factors [103,104].

Oxidative stress further enhances vasoconstriction through endothelin-1 and thromboxane A2, increased hypoxic cytosolic calcium concentration, and reduces vasodilatation through decreased prostacyclin production [105,106,107]. Accordingly, anti-oxidative therapies are beneficial in experimental PAH [108,109]. ROS are produced by inflammatory and vascular cells, and NADPH oxidases are localized in macrophages or polynuclear as well in pulmonary arterial endothelial, smooth muscle cells, and fibroblasts [110,111,112].

Moreover, studies suggested that mtDNA injury participates in PAH development. The mitochondrial Sirtuin 3, involved in mtDNA repair, is decreased in PAH patients and monocrotaline-induced PH in rats [113,114]. 

#### 3.3.2. Mitochondrial Function in Platelets during Pulmonary Hypertension

In PAH, platelets participate in vascular thrombosis through different mechanisms [115,116]. Very interestingly, Nguyen et al. showed that circulating platelets from PAH patient’s exhibit impaired bioenergetics characterized by increased glycolysis compared to healthy controls [81], Table 2. In these patients, increased glycolysis was associated with a switch toward fatty acid oxidation, and increased respiratory reserve capacity correlated with the hemodynamic severity assessed by right heart catheterization. This suggests a relationship between platelet mitochondrial function and PAH severity. In this study, PAH pulmonary vasodilators modulating endothelin, nitric oxide, and/or prostacyclin activities did not affect the mitochondrial bioenergetics observed in PAH platelets, but this deserves to be confirmed in a greater subset of patients.

It might be useful to investigate a potential link between brain natriuretic peptide (BNP), an established biomarker of PAH severity and prognosis, and platelet bioenergetics alteration which could also reflect abnormalities of pulmonary vascular cells or cardiomyocytes and could be useful to assess PAH gravity and progression. Further, since BNP has been shown to protect cardiac and skeletal muscles mitochondria from ischemia-reperfusion damages [117,118], it might be interesting to investigate whether BNP might also modulate circulating cells oxidative capacities.

Interestingly unlike in PAH (group 1), platelets from subjects with HF with preserved ejection fraction (group 2 PH) did not show change in the glycolytic rate compared to normal subjects [82]. However, similarly to PAH patients, they showed an enhanced maximal respiratory capacity. Besides HF, older age and other comorbidities such as obesity, diabetes, or systemic hypertension might explain in part the observed differences in platelet biology.

### 3.4. Idiopathic Pulmonary Fibrosis and Interstitial Lung Diseases

#### Mitochondrial Dysfunction, Oxidative Stress, and Inflammation in Idiopathic Pulmonary Fibrosis

Interstitial lung diseases are characterized by progressive scarring of the lungs. Mechanisms underlying the pathogenesis of these diseases remain incompletely understood and idiopathic pulmonary fibrosis (IPF) is the most common fibrotic interstitial lung disease. Despite anti-fibrotic treatments, IPF has a poor prognosis with a life expectancy of 3–5 years after the diagnosis, which is generally made in patients older than 60 years [3]. A characteristic feature of IPF is the accumulation of myofibroblasts arising from normal lung fibroblasts, largely involved in extracellular matrix remodeling, in response to biochemical courses such as TGF-β (a pro-fibrotic cytokine) or environmental/genetic factors [119]. 

Recent studies suggested a role of mitochondrial dysfunction in term of biogenesis, dynamic (fusion or fission), and mitophagy in the physiopathology of IPF. There is evidence for mitochondrial dysfunction leading to cellular senescence and apoptosis in alveolar epithelial cells (AECs), fibroblasts, and immune cells, participating in the fibrotic process. Besides a potential role of age per se, mitochondrial dysfunction being a recognized hallmark of aging, the alveolar epithelium is sensitive to injury/apoptosis induced by cigarette smoke and other pollutants, which may affect AECs mitochondrial function. TGF-β also regulates the alterations of mitochondrial function [120,121,122].

There is an enhanced mtROS production in fibrotic lungs leading to type II AECs apoptosis, but lung macrophages and fibroblasts are resistant to apoptosis. Mitochondria from fibrotic type II AECs change their shape and become enlarged but mitophagy and biogenesis are reduced. In lung macrophages, altered mitochondria are removed via mitophagy and undergo fission. In fibrotic myofibroblasts, mitochondrial dysfunction has been termed “mitochondrial dysfunction-associated senescence,” characterized by a distinctive senescence-associated secretory phenotype. Fibrotic fibroblasts present with reduced mitophagy and mitochondrial biogenesis but with enhanced fission. MtROS generated at complex III could be responsible for excessive TGF-β in IPF since they are involved in TGF-β mediated gene expression [123].

Regarding these data, antioxidants could be beneficial in IPF. However, a randomized clinical trial testing N-acetylcysteine failed to improve lung function, rate of death, or acute exacerbations ratio in IPF patients, suggesting the role of other associated factors in IPF development [124]. Mitochondrial targeted antioxidant could reduce TGF-β induced pro-fibrotic gene expression and NOX-4 expression, which is necessary for TGF-β induced-myofibroblast differentiation [123].

To date, no work has studied the mitochondrial function in PBMCs or platelets in IPF. A study of circulating fibrocytes oxidative capacity, which are derived from monocytes lineage and thought to be precursors of fibroblasts, could be interesting [125]. Because epidemiological studies showed a link between IPF and cardiovascular diseases or venous thromboembolism, studying mitochondrial function in platelets could also be helpful in these diseases; platelet hyperactivity in patients with IPF being recently demonstrated [126,127].

## 4. Connections between Mitochondrial Dysfunction, ROS, and Inflammatory/Fibrotic Pathways in Lung Diseases. Pathophysiological Hypothesis

**Figure 3:**
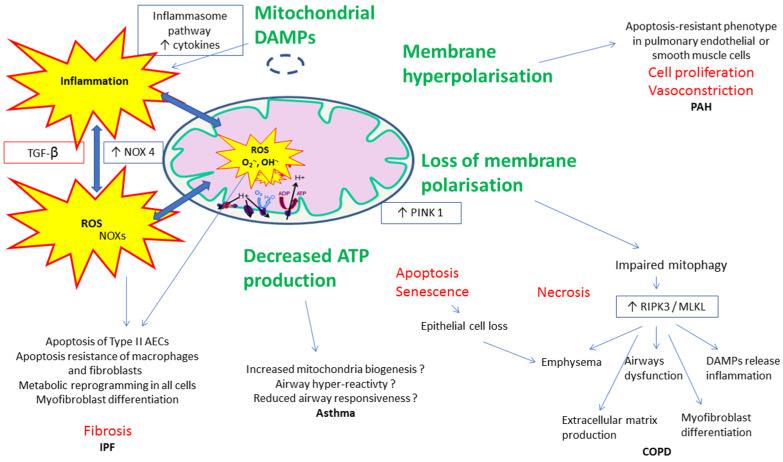
Examples of proposed mechanisms involved in the connections between mitochondrial dysfunction, reactive oxygen species (ROS), and inflammatory/fibrotic pathways in lung diseases.

Such connections are numerous and besides other pathways, mitochondrial membrane polarization changes, altered mitophagy, and damage-associated molecular patterns (DAMPS)-related signaling can be involved in alveolar or pulmonary vascular remodeling observed in lung diseases. For clarity, these pathways are presented separately but they often share mutual mechanisms in COPD, PAH, asthma, and/or IPF (Figure 3).

In PAH, for instance, mitochondrial dysfunction and impaired ATP production promote glycolysis and thus hyperpolarization of the inner mitochondrial membrane, preventing the release of proapoptotic factors and leading to an “apoptosis-resistant” phenotype in pulmonary endothelial or smooth muscle cells. This results in cell proliferation and vasoconstriction [128,129]. Additionally, inhibitors of apoptosis are released from mitochondria when cells are under stress.

In COPD, loss of membrane polarization and elevated expression of the mitophagy regulator protein PINK1 (phosphatase and tensin homolog-induced putative kinase 1), a serine/threonine kinase, in epithelial cells in emphysematous regions of human lungs coincides with increased expression of the protein RIPK3 (receptor-interacting protein kinase 3) which modulates the programmed necrosis and many features characterizing this chronic lung disease [130]. Programmed necrosis (necroptosis) is a cellular program regulated by RIPK1 (receptor-interacting protein kinase 1) and RIPK3, and MLKL (mixed lineage kinase domain-like pseudokinase) in chronic lung diseases.

In asthma, conflicting results have been reported. Thus, decreased ATP production has been reported either to reduce airway constriction which is an energy dependent mechanism, or to be associated with airway smooth muscle thickening [131].

In IPF, the mitochondrial quality control system plays various roles depending on pulmonary cell types. While type II alveolar epithelial cells, lung macrophages, and fibroblasts show increased mtROS production in fibrotic lungs, their response differs. Type II AECs undergo apoptosis but lung macrophages and fibroblasts display apoptosis resistance. In response to increasing oxidative stress, all three cell types undergo a metabolic reprogramming which leads to the development and progression of lung fibrosis. Particularly, fibroblasts are transformed into a myofibroblast state, the effector cells of IPF [132,133,134].

Damage-associated molecular patterns (DAMPs), generated and released by cellular injury, may trigger inflammation, apoptosis, and innate immune responses by activating pattern recognition receptors. Mitochondria-associated DAMPs include mtDNA, which through auto-, paracrine and/or systemic effects activate the inflammasome pathway, resulting in increased cytokine release by immune cells as well resident cells favoring this inflammation, cell proliferation, and apoptosis. Mitochondrial DAMPs can be considered as proinflammatory inductors of the pulmonary remodeling observed in lung diseases and have been correlated to mortality in diseases such as sepsis and IPF. Their precise role in COPD, asthma, or PAH still deserves further studies [29,135].

Of note, ROS do not only arise from mitochondria in lung tissue. The NADPH oxidase homolog NOX4 is overexpressed in the lungs, primarily in myofibroblasts in fibroblastic foci and remodeled blood vessels, but also in epithelial cells associated with aberrant bronchiolization. NOX4 induction is largely mediated by production of the pro-fibrotic growth factor TGF-β. NOX 4 also induces mitochondrial ROS production. The production of ROS promotes mitochondrial DNA damage by reducing the mitochondrial expression of mitochondrial sirtuin 3 and OGG1 to mediate alveolar epithelial cells apoptosis [136,137]. It is the same in PAH, NADPH oxidase homologs NOX 2 and NOX 4 are key producers of ROS in vasculature. Like in IPF, NOX 4 mediates TGF-β1 dependent pulmonary vascular remodeling [138]. NOX 4 also mediates the effect of platelet-derived growth factor (PDGF) and HIF-1α which are critical to the pathogenesis of PAH.

Hypoxia, a frequent condition in lung diseases, induces the production of mitochondrial-derived oxygen-free radicals and ROS by the mitochondrial ETC. Of the molecules involved in this process, H_2_O_2_ can activate the transcription factor and lower oxygen-induced factor HIF-1α, which is implicated in pathophysiology of the lung diseases like PAH. Chen et al. showed that HIF-1α promoted pulmonary arterial smooth muscle cell proliferation and inhibited hypoxia-induced apoptosis, possibly through the regulation of mitochondrial dynamics [139].

Another mechanism which leads to mitochondrial dysfunction in PAH is the nitic oxide (NO)-cyclase guanylate- cyclic GMP pathway. NO regulates cellular respiration and mitochondrial biogenesis. In PAH, decreased NO level is associated with mitochondrial impairment with decreased ATP levels and dysregulated endothelial angiogenesis [140].

## 5. Antioxidative Therapies

Despite a clear involvement of ROS in lung diseases physiopathology, and albeit antioxidants appeared beneficial in experimental models, treatment with antioxidants has been largely unsuccessful and is not part of standard care in humans. Indeed, although improving some functional parameters, N-acetylcysteine or nutrients rich in antioxidants showed generally no beneficial effect on the rate of adverse events or death rates [124,141,142]. The failure of trials might have resulted from an incomplete understanding of the role of mitochondrial ROS in lung diseases development. Indeed, mitochondrial ROS are not always detrimental. They could play a protective role at lower levels and act differently according to the redox microenvironment, which varies spatially and temporally in different subcellular compartments and in different cell types. Thus, only specific subsets of patients might benefit from antioxidant therapies, an individual’s susceptibility potentially depending on variation in their antioxidant genes.

## 6. Conclusions

Mitochondrial dysfunction associated with lung inflammation and oxidative stress contributes to COPD, asthma, PAH, and idiopathic pulmonary fibrosis. To allow adequate function and maintenance of intracellular homeostasis, mitochondria rely on quality control pathways (mitochondrial biogenesis, fusion/fission, mitophagy…), and their alteration disrupts organelle metabolism and biogenesis, inflammation adequacy, and even innate immunity. The general concept that enhanced oxidative metabolism drives toward an anti-inflammatory response and that shifting toward a glycolytic metabolism favor an inflammatory response varies depending on the cells. Mitochondria are also platforms for pattern recognition receptors signal transduction and mediators in effector responses. Thus, mitochondrial DAMPS can activate the NRLP3 inflammasome, resulting in proinflammatory cytokine release. They act as the danger signal recognized by immune receptors [131,143,144]. Accordingly, for instance, TLR9 is involved in mtDNA induced in acute and chronic lung inflammation, through the TLR9-p38 MAPK pathway and STING pathway, respectively [145]. All pulmonary cell types are involved in the triad mitochondrial dysfunction–inflammation–enhanced ROS production.

Besides the role of mitochondrial dysfunction and oxidative stress at the tissue levels, studying PBMCs or the platelet’s redox state takes roots on the fact that these cells play a key role in the inflammatory and immune mechanisms involved in lung diseases. Likely, an altered redox state of dysfunctional circulating blood cells could enhance oxidative stress at the tissue level, potentially worsening the progression of the disease. However, to date, it appears that despite a generally increased ROS production, mitochondria respond quite differently depending on the lung disease. Thus, an impaired mitochondrial oxidative capacity is observed in the case of COPD, but PBMCs and/or platelets mitochondrial respiration is enhanced in the setting of asthma or PH. Whether such stimulated oxidative capacities might be protective, deserves to be determined.

Further studies are needed to reinforce the current evidence and to identify the potential of circulating PBMCs or platelets as biomarkers, which might allow a better understanding of the mechanisms involved in lung diseases, a better follow-up of patients, and possibly might help to identify novel original therapies.

## Figures and Tables

**Table 1:** PBMCs or platelets mitochondria respiration is impaired in chronic obstructive pulmonary disease (COPD).

References	Lung Disease Number of Patients	Type of Circulating Blood Cells	Mitochondrial Respiration	Oxidative Stress	mtDNA	Other Results
De Falco. 2017, Front Immunol [42]	Unstable COPD patients	PBMCs treated with combustion-generated ultrafine particles exposure	High level of mitochondrial dysfunction	High level of mtROS High expression of NOD-like receptor 3 in PBMCs in basal conditions in COPD patients		Release of cytokines: IL-18 and IL-33 (dependent on the release of caspase-4)
Bialas. 2018, Int J Chron Obstruct Pulmon Dis [47]	Chronic smoke-exposed guinea pig	Platelets	Increased proton and electron leak Decreased electron transfer system capacity			
Carpagnano. 2016, BMC Pulm Med [52]	ACOS patients (*n* = 23) COPD patients (*n* = 13) Asthmatic patients (*n* = 14) Normal subjects (*n* = 10)	PBMCs			Increased mtDNA/ nuclear DNA ratio in ACOS patients compared to other groups Increased mtDNA/ nuclear DNA in asthmatic or COPD patients compared to normal subjects	
Agarwal. 2019, Respir Res [43]	Tobacco smoke related COPD patients (*n* = 14) Non-smokers (*n* = 16) Healthy smokers (*n* = 13)	PBMCs	Impaired glucose metabolism in COPD subjects: lower OCR, ATP production, and spare respiratory capacity Impaired pyruvate metabolism in COPD subjects Impaired fatty acid metabolism in COPD subjects			Increase of inflammatory cytokine response (IFN-γ, IL-17, TNF-α, IL-5, IL-9, and IFN-α)
Liu. 2015, PloS One [51]	COPD patients (*n* = 86) Healthy smokers (*n* = 33) Non-smokers (*n* = 77)	PBMCs	Decreased serum glutathione level in COPD		Decreased leukocyte mtDNA copy number of PBMCs in COPD Linear correlation between mtDNA copy number and serum glutathione level	

ACOS: asthma-COPD overlap syndrome; ATP: adenosine triphosphate; COPD: chronic obstructive pulmonary disease; OCR: oxygen consumption rate; PBMCs: peripheral blood mononuclear cells.

**Table 2:** PBMCs or platelets mitochondria respiration is enhanced in asthmatic or in patients with pulmonary arterial hypertension (PAH).

References	Lung Disease Number of Patients	Type of Circulating Blood Cells	Mitochondrial Respiration	Oxidative Stress	mtDNA	Other Results
Ederle. 2019, J Clin Med [67]	Severe asthmatic patients with severe exacerbation (*n* = 20) Healthy volunteers (*n* = 20)	PBMCs	Increased PBMCs mitochondrial respiratory chain complexes activity in asthmatic patients Mitochondrial respiratory chain complexes activity in PBMCs is related to plasma constituent	Increased ROS production in the blood of asthmatic patients ROS production is related to plasma constituent		
Winnica. 2019, Antiox Redox Signal [71]	Lean and obese, mild to moderate, asthmatic patients (*n* = 16) Lean and obese healthy volunteers (*n* = 21)	Platelets	Similar basal OCR in lean healthy and asthmatic subjects Increased basal OCR in asthmatic obese Enhanced maximal OCR in lean and obese asthmatic patients	Enhanced ROS production in lean and obese asthmatics		
Xu. 2015, Plos One. [80]	Asthmatic patients (*n* = 12) Healthy controls (*n* = 13)	Platelets	Similar OCR in both groups Decreased glycolytic rate and greater tricarboxylic acid cycle activity in asthmatic platelets		No change in mtDNA content	No change in mitochondrial number and morphology
Nguyen. 2017, JCI Insight [81]	Group 1 PAH patients (*n* = 28) Control patients (*n* = 28)	Platelets	Increased glycolytic rate in PAH patients: decrease of pyruvate dehydrogenase activity No change in basal respiration Enhanced respiratory reserve capacity in PAH dependent on increased fatty acid oxidation Increase in complex II enzymatic activity and decrease in complex I enzymatic activity. No change in enzymatic activity of complex IV.	No change in mitochondrial superoxide production		Positive correlation between respiratory reserve capacity and hemodynamic severity (mean PAP, PVR and right ventricle stroke work index) No change following phosphodiesterase 5 inhibitions, prostacyclin analogue and endothelin receptor antagonist
Nguyen. 2019, Plos one. [82]	Group 2 PH patients (*n* = 20) Control patients (*n* = 20)	Platelets	No significant difference in basal oxygen consumption rate. Increased maximal oxygen consumption rate (increased contribution of fatty acid and glucose oxidation).	No difference in mitochondrial superoxide production.		Negative correlation between maximal mitochondrial respiration and right ventricular stroke work index No change following nitrite inhalation.

ETC: electron transport chain; OCR: oxygen consumption rate; PAH: pulmonary arterial hypertension; PAP: pulmonary arterial pressure; PVR: pulmonary vascular resistance.
